# Editorial: Transfusion medicine and blood, volume II

**DOI:** 10.3389/fmed.2024.1428574

**Published:** 2024-05-21

**Authors:** Robert W. Maitta, Magali J. Fontaine, Hollie M. Reeves, Ljiljana V. Vasovic, Laura Infanti

**Affiliations:** ^1^Department of Pathology, Case Western Reserve University, Cleveland, OH, United States; ^2^Department of Pathology, University of Maryland, Baltimore, MD, United States; ^3^Department of Pathology and Laboratory Medicine, Nationwide Children's Hospital, Columbus, OH, United States; ^4^Pathology and Laboratory Medicine, New York Medical College, Valhalla, NY, United States; ^5^Laboratory Medicine/Hematology, University Hospital of Basel, Basel, Switzerland

**Keywords:** transfusion, platelets, components, red blood cells, donations

Transfusion of blood components represents the most commonly used therapeutic approach to treat patients presenting with anemia, cytopenias and even coagulopathies to alleviate the symptomatology associated with a given deficiency. In light of this, it should come as no surprise that transfusion medicine represents the field that affects practically all specialties in medicine. Indeed, over the past decades the field has moved to a highly structured scientific discipline pioneering new developments that have changed how and when blood components are used, while achieving greater understanding of their biology. Importantly, evidence-based approaches set forth by a growing number of professional societies have resulted in new guidelines that aim to bring about a judicious use of the existing precious blood supply.

Those of us in the field know that current levels of blood donations, though addressing the needs of patients, has brought attention to the reality and challenges of a shrinking donor pool (1, 2). Testing for infectious agents to guarantee a safer blood supply is paramount not only to maintain but to expand this supply further while continuing to improve its safety. In this Research Topic, a report from Colombia describes the likelihood of arbovirus infections in blood donors by comparing viral testing results during epidemics to pandemic time-periods (Cáceres Munar et al.). Their data demonstrate that the majority of those who tested positive for one of the arboviruses' nucleic acids under study were completely asymptomatic; and for those donors with symptoms these were non-specific such as headache, fever, arthralgias, or malaise. Notably, the rate of infection and of asymptomatic infections was far greater than prior published data and argues for proactive, vigilant, and revamped screening processes of donors. Looking at other developing regions of the world, a report from sub-Saharan Africa describes the promising results of the German Federal Ministry of Health's BloodTrain program to assist blood regulatory agencies in sub–Saharan African countries to improve their guidelines and recommendations for the use of *in vitro* point of care medical devices for donor screening. The aim of this program is to increase blood inventory through improved and well-regulated testing platforms across the region (Abdurrahman et al.). Both of these manuscripts will certainly be of broad interest to the practitioners and the transfusion medicine community.

Continuing along the lines of blood safety, determining which donors are suitable for donation and finding ways to improve overall donor health are essential to a sustainable blood supply. Thus, a report in this Research Topic from China describing how donor deferrals were used to establish trends in donors' health is significant (Feng et al.). This study indicates that abnormal vital signs and laboratory values such as low hemoglobin concentration and high liver transaminases point at reasons for the chronological increase in the number of deferrals and that these may be influenced by age, gender and diet among other factors. In contrast, a report seeking to determine the demographic characteristics of blood donors in Poland indicated that these tend to be married males with at least a high school education (Siekierska et al.). This study emphasizes that increases in the blood donor pool will require a change in the health education and information provided to students so their level of understanding of blood donations encourages them to participate. Lastly, a report from Central Europe looking at blood obtained from hemochromatosis patients as sources of donations indicates that use of erythrocytapheresis for controlling iron and ferritin was more effective in reduction of both variables than regular phlebotomies (Infanti et al.). Surprisingly, this study indicates that these interventions to reduce iron may have the unintended effect to lower hemoglobin concentration and to cause fatigue in the setting of developing anemia. The authors suggest that this approach should be carefully evaluated to minimize complications of chronic red blood cell reduction in hemochromatosis. Indeed, the procedures themselves may need to be spaced out more or canceled altogether due to these adverse events.

In summary, this Research Topic of articles detailing how different regions and countries around the world maintain a safe and sustainable blood donor pool and blood supply will inform while simultaneously raising awareness of the challenges encountered by transfusion medicine professionals and blood centers alike. Readers of this Research Topic and services who depend on blood components as therapies for their patients will find the Research Topic to be balanced and up to date, while it challenges us to become active participants in improving blood collections with an emphasis on quality and safety.

## Author contributions

RM: Conceptualization, Project administration, Writing—original draft, Writing—review & editing. MF: Writing—review & editing. HR: Writing—review & editing. LV: Writing—review & editing. LI: Writing—review & editing.
